# Mechanisms and therapeutic strategies of extracellular vesicles in cardiovascular diseases

**DOI:** 10.1002/mco2.454

**Published:** 2023-12-20

**Authors:** Qirong Li, Qiang Feng, Hengzong Zhou, Chao Lin, Xiaoming Sun, Chaoyang Ma, Liqun Sun, Gongliang Guo, Dongxu Wang

**Affiliations:** ^1^ Department of Cardiology China‐Japan Union Hospital of Jilin University Changchun China; ^2^ Laboratory Animal Center College of Animal Science Jilin University Changchun China; ^3^ School of Grain Science and Technology Jilin Business and Technology College Changchun China; ^4^ Hepatology Hospital of Jilin Province Changchun China; ^5^ Department of Pathogenobiology Jilin University Mycology Research Center College of Basic Medical Sciences Jilin University Changchun China

**Keywords:** cardiovascular disease, exosomes, extracellular vesicles, myocardial injury, stem cells

## Abstract

Cardiovascular disease (CVD) significantly impacts global society since it is the leading cause of death and disability worldwide, and extracellular vesicle (EV)‐based therapies have been extensively investigated. EV delivery is involved in mediating the progression of CVDs and has great potential to be biomarker and therapeutic molecular carrier. Besides, EVs from stem cells and cardiac cells can effectively protect the heart from various pathologic conditions, and then serve as an alternative treatment for CVDs. Moreover, the research of using EVs as delivery carriers of therapeutic molecules, membrane engineering modification of EVs, or combining EVs with biomaterials further improves the application potential of EVs in clinical treatment. However, currently there are only a few articles summarizing the application of EVs in CVDs. This review provides an overview of the role of EVs in the pathogenesis and diagnosis of CVDs. It also focuses on how EVs promote the repair of myocardial injury and therapeutic methods of CVDs. In conclusion, it is of great significance to review the research on the application of EVs in the treatment of CVDs, which lays a foundation for further exploration of the role of EVs, and clarifies the prospect of EVs in the treatment of myocardial injury.

## Graphical abstract

Extracellular vesicles (EVs) have many potential applications in the study of cardiovascular pathophysiology. This article reviews the role of EVs in the pathogenesis and diagnosis of cardiovascular diseases (CVDs) in cardiovascular system. In addition, the mechanism and therapeutic methods of EVs promoting CVDs repair were summarized.

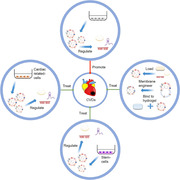

## 1 INTRODUCTION

The incidence and mortality of cardiovascular diseases (CVDs) remain high worldwide.^1^ CVDs include atherosclerosis, ischemic heart disease, cerebrovascular disease, hemorrhagic stroke, hypertensive heart disease, cardiomyopathy, myocarditis, atrial fibrillation (AF), aortic aneurysm, peripheral vascular disease, and endocarditis.^2^ Mammalian cardiomyocytes have a limited capacity to proliferate after cardiac injury. Therefore, myocardial damage in CVDs is a major factor leading to adverse fibrosis and myocardial remodeling, contributing to the development of cardiac dysfunction and, ultimately, to heart failure (HF).^3^ The pathogenesis of CVDs is complex due to many factors. Cardiac injury (including stress overload‐induced HF and ischemic injury) often leads to cardiac fibrosis, where a reparative fibrosis scar replaces heart tissue and gradually reshapes the tissue during myocardial cell death.^4^ Several therapeutic interventions have been previously proposed, and cell‐based therapies are currently being intensively investigated.^5^ However, the poor survival rate of transplanted cells in the ischemic heart tissue environment limits the clinical efficacy of cell therapy.^6^ There is an urgent need to develop effective myocardial repair therapies and to clarify the detailed mechanisms of cardiac repair after CVDs.

Almost all known cell types secrete extracellular vesicles (EVs), which have the lipid bilayer structure.^7^ EVs can be broadly classified into three types: apoptotic bodies, microvesicles, and exosomes.^8^ Exosomes are small membrane‐bound vesicles (30–100 nm) that are produced during endosomal maturation into multivesicular bodies.^9^ Microvesicles are small vesicles (100–1000 nm in diameter) that are shed from the cell membrane after cell activation, injury, or apoptosis. They originate from the cavitary membrane of polyvesicles and are released via the fusion of polyvesicles with the cell membrane. Under both normal and pathological conditions, EVs facilitate micro‐communication at the levels of cells, tissues, and organs by transporting proteins, mRNAs, and microRNAs (miRNAs/miRs).^10^ Further research has identified many functions for EVs, including involvement in immune responses, antigen presentation, tumor cell migration and proliferation, and regulation of apoptosis and autophagy.^11^

Many studies have also shown that EVs play an emerging role in the context of CVDs.^12^ The heart comprises diverse groups of cells, such as cardiomyocytes, fibroblasts, pericytes, smooth muscle cells, endothelial cells (ECs), and various immune cells,^13^ which can play a role in repairing CVDs through cell‐to‐cell communication pathways such as the secretion of EVs. In addition, multiple studies have shown the potential involvement of circulating or endogenous stem cells in restoring myocardial tissue following irreversible injury.^14^ Cell‐free treatments based on EVs have attracted the interest of many researchers because they avoid the need to transplant large numbers of cells and are easier to apply. In addition, combining bioengineering methods with EVs to improve their delivery rate and stability in vivo is also an emerging research area in CVDs. Improving EV application through engineering methods may enhance their clinical potential in CVD applications.^15^ In particular, hydrogels have been used as excellent carriers and can be used as cardiac patches or stents to achieve local delivery of therapeutic molecules and improve EV retention.^16^

This review summarizes the EVs that promote the pathogenesis of CVDs and can be used as diagnostic markers. The regulatory mechanism of EVs from different cardiac or stem cells promoting the repair of CVDs is also introduced. In addition, the therapeutic potential and bioengineering strategies of EVs in CVDs are further described. Moreover, it provides evidence for future clinical treatment of patients with CVDs. It also clarifies the therapeutic effect and potential molecular mechanism of CVDs treatment with EVs to a certain extent.

## 2 EVs AND CVDs

**FIGURE 1:**
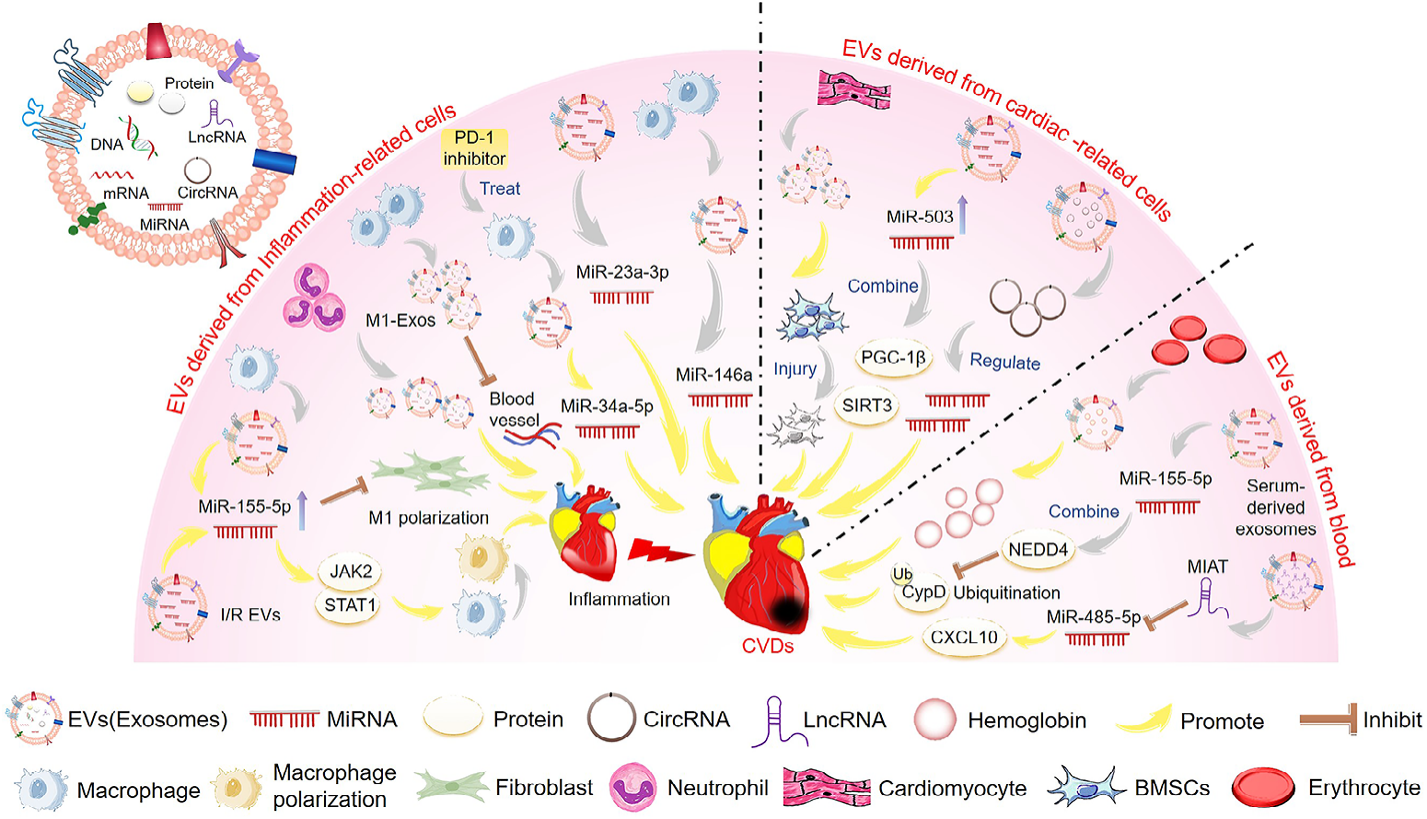
Mechanisms of extracellular vesicles (EVs) in the pathogenesis of cardiovascular diseases (CVDs). EVs from inflammation‐related cells influence the progression of CVDs by inhibiting macrophage polarization, angiogenesis, and promoting inflammatory responses. EVs from cardiac‐related cells influence CVDs progression through regulation of mesenchymal stem cells (MSCs) and delivery of microRNAs (miRNAs) and circular RNAs (circRNAs). EVs from platelets and serum also play a role in promoting CVDs. The structure schematic diagram of EVs is in the upper left corner. EVs usually contain substances such as DNA, RNA, and proteins. The yellow arrow represents the process of promoting CVDs.

EVs are nanoscale structures secreted by cells that contain various biomolecules involved in cellular communication and physiological processes.^17^ Increasing evidence confirms the important role of EVs as a tool for intercellular communication.^18^ EVs have attracted considerable attention due to their unique properties, making them an ideal choice for diagnosis and treatment. EVs, including exosomes, are an early marker of CVDs and a key mediator of cardiac protection.^19^ In addition, many studies have shown that non‐coding RNAs (ncRNAs) play an important role in CVDs. Moreover, EVs can be involved in pathogenesis by transferring relevant regulatory biomolecules, such as ncRNAs, to target cells to regulate their function and activate or silence genes^20^ (Figure 1).

### 2.1 The role of EVs in the pathogenesis of CVDs

Acute myocardial infarction (AMI) is a common manifestation of ischemic heart disease/coronary artery disease (CAD) with significant morbidity and mortality, posing a challenge worldwide.^21^ Coronary reperfusion can lead to irreversible myocardial damage, known as myocardial ischemia/reperfusion (I/R) injury.^22^ Myocardial I/R‐induced myocardial cell damage, such as apoptosis and hypertrophy, can aggravate cardiac insufficiency and increase the incidence of poor prognosis. In addition, hypoxia–reoxygenation (H/R) injury can cause various pathophysiological alterations in the myocardium, potentially resulting in the development of myocardial energy metabolism disorders and microvascular dysfunction.^23^ H/R conditions are usually used to simulate I/R injury in myocardial cells.

Inflammation is a fundamental biological process that helps to maintain homeostasis, and the initial inflammatory response following a myocardial infarction (MI) is associated with the eventual size of the affected area.^24^ Macrophages are important effectors and regulators of the inflammatory cascade induced by MI. The polarization of macrophages during MI and I/R myocardial injury can be a potential therapeutic target.^25^ I/R‐EVs aggravated I/R‐induced heart injury, promoted the M1‐like polarization of macrophages, and increased the expression of proinflammatory cytokines. In addition, other studies found that I/R‐EVs could deliver miR‐155‐5p to macrophages and activate the Janus kinase 2 (JAK2)/signal transducer and activator of transcription 1 (STAT1) pathway, enhancing the inflammatory response and exacerbating local inflammation in the heart.^26^ In addition, miR‐34a‐5p was identified as an exosome transferable molecule that induces cardiac aging‐related damage. The exosomes of macrophages treated with programmed cell death 1 (*PDCD1*/*PD‐1*) inhibitors can upregulate miR‐34a‐5p in cardiomyocytes and cause cardiac injury in mice. The results may provide new therapeutic targets for improving myocardial damage in cancer patients treated with PD‐1 inhibitors.^27^ Moreover, studies have shown that M1‐like macrophages release many proinflammatory exosomes after MI, which have an anti‐angiogenic role and accelerate MI injury.^28^ Additionally, miR‐155 in exosomes released by macrophages inhibits fibroblast proliferation, promotes inflammation at the cardiac injury site, and aggravates the adverse effects of myocardial injury.^29^ Neutrophils will rapidly accumulate in the ischemic area of the heart after MI and trigger the inflammatory response, form an extracellular trap, and produce EVs to exacerbate inflammation and heart damage.^30^ Therefore, EVs containing inflammation‐related factors are involved in important pathways regulating CVDs.

Studies have shown that I/R significantly increases EV release by heart cells.^31^ It has been shown that circular RNA (circRNA) levels in EVs isolated from mouse hearts were altered after I/R myocardial injury. The RNA sequencing of EVs identified 185 circRNAs whose levels differed significantly. circRNA–miRNA analysis showed that these circRNAs could regulate target genes by regulating miRNAs, providing some potential targets and pathways involved in I/R injury.^31^ Other studies have found that exosomes from damaged cardiomyocytes damaged and decreased the survival of transplanted bone marrow mesenchymal stem cells (BMSCs) for heart infarction, suggesting a new mechanism for cell survival after MI.^32^ In addition, EVs induced by AMI impaired myocardial cell viability and worsened the extent of cardiac injury. miR‐503 is abundant in EVs during the early stages of AMI and plays a critical role in mediating myocardial injury. It induces mitochondrial metabolic dysfunction and myocardial cell death by interacting directly with peroxisome proliferator‐activated receptor γ‐co‐activator 1β (*PGC‐1β*) and mitochondrial deacetylase sirtuin 3 (*SIRT3*).^33^ Other studies have shown that miR‐155‐5p in serum‐derived exosomes can promote myocardial I/R injury by reducing peptidylprolyl isomerase F (PPIF/CypD) ubiquitination by neural precursor cell expressed, developmentally downregulated 4 (*NEDD4*).^34^ In addition, erythrocyte and cardio‐derived exosomal hemoglobin might be associated with myocardial injury in patients with valvular heart disease, potentially contributing to acute cardiac damage in humans and animal models.^35^

AF is a global epidemic with significant morbidity, mortality, and socio‐economic burden.^36^ Studies have shown that EVs from the epicardial fat of AF patients are proinflammatory, profibrotic, and arrhythmogenic, thus promoting AF.^37^ In addition, serum‐derived EVs containing long ncRNA (lncRNA) MIAT promote atrial fibrosis, inflammation, and oxidative stress by eliminating miR‐485‐5p‐mediated C–X–C motif ligand 10 (CXCL10) inhibition, and AF.^38^ Atherosclerosis is a chronic inflammatory disease characterized by the formation of atherosclerotic plaques in the lining of the arteries and is a major cause of CAD, stroke, heart attack, and peripheral vascular disease.^39^ EV‐derived miRNAs from atherogenic macrophages, in particular miR‐146a, may accelerate the development of atherosclerosis by reducing cell migration and promoting macrophage capture in blood vessel walls.^40^ Most cells involved in atherosclerosis release EVs, which can carry bioactive substances to downstream tissues through circulation. Studies have shown that EVs may transfer atherosclerosis to remote locations by carrying proinflammatory factors, specifically miR‐23a‐3p.^41^ In addition, EVs released after monocyte/platelet activation may play a potential role in the development and progression of atherosclerosis, exhibiting proinflammatory effects.^42^

Therefore, inflammation‐related cells, including macrophages and neutrophils, mainly deliver miRNAs and inflammation‐related factors through EVs to promote inflammation of CVDs. In addition, cardiogenic EVs isolated from damaged myocardium or serum are mainly involved in the pathway of CVDs through the regulation of circRNAs and miRNAs.

### 2.2 Potential role of EVs in diagnosing CVDs

Current methods for diagnosing CVDs are relatively expensive and fail to detect CVDs at an early stage. New biomarkers with diagnostic and risk assessment potential for CVDs are urgently needed (Table 1). EVs are beneficial for liquid biopsies due to their ability to circulate in the body and their assessable contents.^43^ Besides, levels of certain miRNA biomarkers extracted from EVs have been recognized as promising indicators for early CVD detection.^44^

**TABLE 1:** Extracellular vesicles (EVs) as an indicator in the diagnosis of myocardial injury is under preclinical study.

Disease	Source of EVs	EVs contents as markers	Effects	Reference
Myocardial ischemia/reperfusion injury	Ischemic myocardium	Cardiac and muscle‐specific microRNAs	As a specific early biomarker of heart injury	^47^
Acute myocardial infarction	Urine and blood	The expression levels of miR‐1 and miR‐208 were increased	As a novel urine biomarker for acute myocardial infarction	^48^
Myocardial infarction	Plasma	APOD, APOC3, C1Q1A, C5, GP1BA, PPBP	Protein markers that can be used as biomarkers of myocardial injury	^49^
Doxorubicin‐induced cardiac injury	Cardiomyocyte	Glycogen phosphorylase B	Protein biomarkers of early heart injury	^50^
Heart failure	Cardiomyocytes and endothelium	Trans‐coronary EVs concentration gradients	As a potential biomarker associated with pathophysiology in heart failure	^52^
Systemic inflammatory response syndrome	Cardiomyocyte	Number of cyclic EVs	Reflects cardiac damage in lipopolysaccharide‐induced systemic inflammation	^53^
Non‐valvular atrial fibrillation	Blood	miR‐106b‐3p, miR‐590‐5p, miR‐339‐3p, miR‐378‐3p, miR‐328‐3p, and miR‐532‐3p	Develop the novel biomarkers for diagnosis or monitoring the patients with the high risk of atrial fibrillation	^54^

EVs carrying specific miRNAs have demonstrated their potential as diagnostic and prognostic markers in various clinical settings. The presence of exosomes in the plasma of critical patients suggests their potential utility in both diagnosis and treatment.^45^ Circulating miRNAs released by the damaged myocardium after MI can be transported to distant organs via exosomes.^46^ Plasma circulating miRNAs are highly specific to heart injury and can be used as biomarkers for early diagnosis of MI. Research has shown that EVs are discharged from the myocardium during I/R injury, carrying heart‐ and muscle‐specific miRNAs that can be rapidly identified in plasma. Because these EVs are rich in miRNAs and their detection precedes conventional injury markers, they have great potential as specific early biomarkers of myocardial injury.^47^ miR‐1 and miR‐208 levels were significantly elevated in the urine of patients with AMI and the blood exosomes of rats after AMI and could be used as novel urine biomarkers.^48^ In addition, six proteins belonging to lipid metabolism (apolipoproteins D [APOD] and C3 [APOC3]), complement activation (complement C1q subcomponent subunit A [C1QA] and C5), and platelet activation pathway (glycoprotein Ib platelet subunit alpha [GP1BA] and pro‐platelet basic protein [PPBP]) were identified as biomarkers of myocardial injury in proteomics analysis of plasma‐derived EVs from patients with MI. These findings suggest that EVs in the plasma show promise as a novel biomarker for diagnosis and as a therapeutic target, which could be refined for future clinical use.^49^ Moreover, serum EVs extracted from a mouse model of doxorubicin (DOX)‐induced heart injury showed that those released by cardiomyocytes contained glycogen phosphorylase B (PYGB), a protein biomarker of early heart injury.^50^

HF is a common and severe clinical condition that arises when the heart fails to supply sufficient blood flow to meet the body's metabolic needs or receive systemic venous return. HF is caused by myocardial damage due to various factors, including ischemic heart disease, hypertension, and diabetes.^51^ Due to HF's intricate clinical and mechanistic nature, investigating biomarkers that may provide crucial pathophysiological information and therapies to mitigate cardiac injury is of utmost importance. EVs have also been shown to be a potential biomarker associated with HF pathophysiology. In patients with HF phenotypes undergoing elective coronary artery bypass surgery, EV release curves from different cell sources might indicate myocardial injury and stress states.^52^ Additionally, EVs originating from myocardial cells can be found in the bloodstream. The elevated levels of cardio‐derived EVs in the circulation indicate cardiac injury in the context of lipopolysaccharide‐induced systemic inflammation.^53^ Non‐valvular AF is the most common type of arrhythmia. Compared with the non AF control group, there are six highly expressed miRNAs in EVs of AF patients (miR‐106b‐3p, miR‐590‐5p, miR‐339‐3p, miR‐378‐3p, miR‐328‐3p, and miR‐532‐3p), which may become new biomarkers for diagnosing or monitoring high‐risk AF patients.^54^

In summary, EVs play an important role in the study of the pathogenesis and diagnostic markers of CVDs. In addition, further research on the therapeutic effects of EVs from different cell sources on CVDs is also of great significance.

## 3 EVS DERIVED FROM DIFFERENT CELL TYPES PROMOTE THE TREATMENT OF CVDS

Research has shown that the myocardial tissue can release EVs, which could be a significant means of intercellular communication beyond the confines of the heart.^55^ EVs are submicron‐sized lipid envelopes that are produced and released from parent cells and can be taken up by recipient cells. EVs are able to mediate cell signaling by carrying nucleic acids, proteins, lipids, and cellular metabolites between cells and organs,^56^ and the endogenous properties of these vesicles allow them to survive in the extracellular space, bypass biological barriers and deliver their bioactive molecular cargo to recipient cells.^15^ EVs from non‐cardiac cells and platelets in the circulation, and those from various cardiovascular cell types, may specifically target cardiovascular structures where they can mitigate myocardial injury, while EVs of cardiovascular origin may also be cyclically transferred to distant tissues.^57^ Moreover, previous studies have shown that when mesenchymal stem cells (MSCs) are transplanted into the heart after AMI or I/R injury, infarct size and cardiac function improved, accompanied by a decrease in myocardial cell death.^58^ However, using MSCs during post‐ischemic cardiac functional recovery has great uncertainty and controversial efficacy, limiting their application prospects, which the paracrine properties of MSCs can overcome. Many studies have used modified MSCs and MSC‐EVs to treat myocardial injury and achieved significant results.^59^ As an alternative to cell therapy, EVs have made significant research progress. Studying EVs can help explore the communication between the damaged myocardium and other tissues and organs to initiate the myocardial repair process. In addition, the potential mechanisms of how cardiomyocytes/stem cells repair and regenerate myocardial injury after CVDs can be further elucidated.

### 3.1 Mechanism of cardiovascular cell‐derived EVs in treating CVDs

#### 3.1.1 EVs derived from cardiomyocytes

**FIGURE 2:**
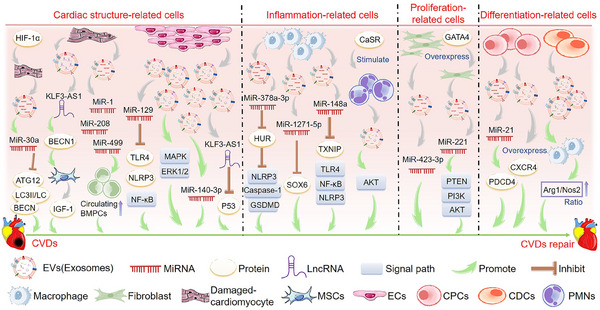
The main mechanism of extracellular vesicles (EVs) derived from different kinds of cardiac cells to promote the repair of cardiovascular diseases (CVDs). EVs derived from cardiac structure‐related cells, such as damaged‐cardiomyocyte and endothelial cells (ECs), promote the repair of CVDs by regulating related signaling pathways. EVs derived from inflammation‐related cells, such as macrophage and polymorphonuclear cells (PMNs), promote the repair of CVDs by regulating related signaling pathways. EVs derived from proliferation‐related cells, such as mesenchymal stem cells (MSCs), promote the repair of CVDs by regulating related signaling pathways. EVs derived from differentiation‐related cells, such as cardiac progenitor cells (CPCs) and cardiosphere‐derived cells (CDCs), promote the repair of CVDs by regulating related signaling pathways. The green arrow represents the promotion of CVDs repair.

Cardiomyocytes, ECs, macrophages, and fibroblasts are the primary sources of EVs in the heart^60^ (Figure 2). Studies have shown that AMI temporarily increases the production of cardiac EVs, and EVs accumulated in ischemic myocardium are rapidly absorbed by infiltrated monocytes and regulate local inflammatory responses.^61^ Besides, EVs from human heart‐derived adherent proliferative (CardAP) cells can direct CD14 immune cells to a regulated type that promotes regeneration of damaged heart tissue by limiting unnecessary inflammatory processes.^62^ The transcription factor hypoxia‐inducing factor 1α (HIF‐1α) plays a crucial role in the adaptive response to hypoxia since it is stimulated by low oxygen levels. Myocytes reportedly release miR‐30a‐rich EVs in response to HIF‐1α to protect the myocardium from damage. miR‐30a‐rich EVs have also been associated with the mechanism of action of downregulating autophagy‐related proteins, such as autophagy‐related 12 (*ATG12*), beclin 1 (*BECN1*), and *LC3II*/*LC3I*.^63^ In addition, exosomal lncRNA KLF3 antisense RNA 1 (*KLF3‐AS1*) from ischemic cardiomyocytes can rescue myocardial cell damage in vitro and in vivo and inhibit myocardial I/R damage by upregulating signal transducer and activator of transcription 5B (*STAT5B*) expression and mediating insulin‐like growth factor 1 (IGF‐1) secretion by MSCs.^64^ It has also been shown that myocardial miRNA (myo‐miR) is released into circulation via exosomes after AMI, mediating functional crosstalk between the ischemic heart and bone marrow (BM). Circulating levels of myo‐miR were elevated in a mouse model of AMI. In addition, the circulating exosomes in this model primarily contained miR‐1, miR‐208, and miR‐499, which can downregulate C–X–C motif chemokine receptor 4 (*CXCR4*) expression in BM monocytes, leading to an increase in the number of circulating progenitor cells (PCs). Therefore, the myo‐miR transported by circulating exosomes can stimulate a systemic response to cardiac injury and has potential applications in cardiac repair.^65^

#### 3.1.2 EVs derived from ECs

ECs comprise the innermost layer of the cardiovascular system and play a crucial role in maintaining the structure and function of the heart and blood vessels. Studies have shown that EVs released by ECs can reduce myocardial cell death after H/R by activating the extracellular signal‐regulated kinases 1/2 (ERK1/2)–mitogen‐activated protein kinase (MAPK) signaling pathway, suggesting that EC‐EVs might have the potential to improve tissue damage in different CVDs.^66^ Another study showed that EVs derived from human umbilical vein ECs could carry miR‐129 and reduce myocardial I/R damage by downregulating Toll‐like receptor 4 (*TLR4*) and disrupting nuclear factor kappa B (NF‐κB) signaling.^67^ Other studies have found that EC‐EVs contain various protective proteins, which may save I/R damage in the human heart‐on‐a‐chip by providing proteins regulating metabolism and rescue pathways to injured cardiomyocytes.^68^ In addition, vascular ECs produce exosomal long intergenic non‐protein coding RNA 174 (*LINC00174*), which can suppress p53‐mediated autophagy and apoptosis to alleviate I/R injury. These findings suggest that targeting *LINC00174* could be promising for treating I/R‐induced MI.^69^ Both internal and external factors can influence the secretion of exosomes or their contents. Notably, shockwave therapy has been shown to stimulate the release of exosomes.^70^ Extracorporeal cardiac shock wave (ECSW) represents a novel, non‐invasive, safe, and effective approach to managing CVDs. Studies have shown that ECSW can effectively stimulate endothelial colony‐forming cell‐derived exosomes in vitro. The delivery of exosomal miR‐140‐3p induced by ECSW can lead to a more potent therapeutic effect on H9C2 cells following H/R‐induced injury, which may represent a promising strategy for treating myocardial IR injury.^71^

#### 3.1.3 EVs derived from the epicardium

The epicardium is the outermost layer of the heart. It can provide various cardiovascular cells for the formation of the heart and is the source of nutritional signals that promote the growth of the heart muscle during embryonic development.^72^ Epicardial EVs have been isolated from mouse and human sources, showing that epicardial EVs act as signaling molecules, promoting the proliferation of cardiomyocytes in mouse hearts with infarction and in models of human myocardial injury, thereby enhancing cardiac function. Moreover, next‐generation RNA sequencing showed conserved miRNAs in the human and mouse epicardial exosomes, such as miR‐30a, miR‐100, miR‐27a, and miR‐30e.^73^

#### 3.1.4 EVs derived from macrophages and polymorphonuclear cells

Studies have shown that EVs derived from M2 macrophages can inhibit ELAV‐like RNA binding protein 1 (*ELAVL1*/*HuR*) expression via miR‐378a‐3p and block the activation of NLR family pyrin domain containing 3 (NLRP3)/caspase 1 (CASP1)/gasdermin D (GSDMD) pathway, thereby reducing myocardial pyrodeath, and restoring cardiac function in mice with MI.^74^ Additional studies have shown that exosomes derived from M2 macrophages contain miR‐148a, which can mitigate myocardial I/R injury by suppressing the thioredoxin interacting protein (TXNIP) and TLR4/NF‐κB/NLRP3 inflammasome signaling pathways.^75^ In addition, M2‐macrophage‐derived exosomes contain miR‐1271‐5p, which can alleviate cardiac damage in AMI by downregulating SRY‐box transcription factor 6 (*SOX6*).^76^ Moreover, polymorphonuclear cells (PMNs) are important inflammatory cells, and studies have shown that calcium‐sensitive receptors (CaSRs) regulate the proinflammatory effects of PMNs. Studies have shown that PMN‐derived exosomes from CaSR stimulation play a pivotal role in AMI models and myocardial reperfusion injury. This effect may be associated with the protein kinase B (AKT) signaling pathway.^77^

#### 3.1.5 EVs derived from fibroblasts

The cardioprotective effects of post‐ischemic regulation are mediated by exosomal miR‐423‐3p originating from cardiac fibroblasts, protecting cardiomyocytes from H/R damage.^78^ Moreover, overexpression of GATA binding protein 4 (*GATA4*) enhanced the protective effects of exosomes secreted by cardiac colony‐forming unit fibroblasts against myocardial ischemic injury via the miR‐221‐mediated phosphatase and tensin homolog (PTEN)/phosphoinositide 3‐kinase (PI3K)/AKT signaling pathway.^79^

#### 3.1.6 EVs derived from regeneration‐associated cells

Discovering novel approaches for converting cardiomyocytes into a regenerative state is a significant therapeutic objective, given that the heart has limited regenerative potential to repair damaged tissue following a MI, which may subsequently result in additional HF.^80^ When a myocardial injury requires angiogenesis, proinflammatory cell subsets of peripheral blood monocytes change their phenotype to pro‐regenerative cells, such as angiogenic endothelial PCs, M2 macrophages, and regulatory T cells (Tregs), collectively known as regeneration‐associated cells (RACs).^81^ EVs secreted by RACs contribute to angiogenesis, anti‐fibrosis, anti‐inflammation, and cardiomyogenesis. They can preferentially accumulate in the I/R‐injured myocardium and contribute to the recovery of cardiac function after ischemia‐induced injury.^82^

#### 3.1.7 EVs derived from cardiac PCs

Cardiac PCs (CPCs) are stationary cells under normal physiological conditions, but they are activated and have the potential to differentiate into cardiac cells after cardiac injury. Treating mouse models of I/R injury and mouse myocardial cells with CPC‐derived EVs reduced myocardial cell apoptosis.^83^ In addition, EVs from human CPCs suppressed myocardial cell apoptosis and enhanced cardiac function post‐injury.^84^ In vitro and in vivo studies have shown that EVs derived from CPCs can ameliorate cardiac function and reduce fibrosis. This protective effect of cardiomyocytes is mediated by post‐transcriptional regulation of miR‐21 by *PDCD4*.^85^ Elevated *CXCR4* levels in CPC‐derived exosomes has been shown to improve the efficacy and bioavailability of cardio‐protective CPC exosomes and improve cardiac function in rat models with I/R injury.^86^ Additionally, the migration of PCs and other supportive cells from the BM to the ischemic injured heart is a natural reparative response. Clinical trials of cell therapy using BM‐PCs have demonstrated its therapeutic effect on ischemic heart disease.^87^

#### 3.1.8 EVs derived from cardiosphere‐derived cells

Several studies have indicated that using exosomes released by cardiosphere‐derived cells (CDCs) may serve as a viable cell‐free therapy for CVDs. Arrhythmogenic cardiomyopathy (ACM) is an inherited heart disease characterized by progressive loss of cardiomyocytes and replacement of fibrous adipose tissue.^88^ It has been shown that EVs derived from CDCs mitigated ACM in desmoglein‐2 mutant mice, improved heart function, reduced cardiac inflammation, and inhibited the occurrence of arrhythmias.^89^ Moreover, in two distinct porcine models, the acute administration of CDC‐derived exosomes reduced myocardial injury, reduced scar formation, mitigated adverse cardiac remodeling, and improved function in both acute and chronic MI phases. These findings support the potential use of CDC‐derived exosomes as a promising therapeutic strategy for MI.^90^ Furthermore, studies have shown that intravenous CDC‐EVs accumulate primarily in the heart and that cardiac injury enhances the uptake of CDC‐EVs by the heart, liver, and brain.^91^ Additional studies have proposed that CDCs might be more efficacious than MSCs in MI models. In vitro, CDC‐derived EVs have been shown to elevate the arginase 1 (*ARG1*)/nitric oxide synthase 2 (*NOS2*) ratio in macrophages, leading to a greater reduction in MI size than MSC‐derived EVs. Moreover, in animal models, CDC‐derived EVs have shown protective effects against ischemic myocardial injury and acute inflammation.^92^

In summary, EVs derived from various sources can promote the repair of CVDs. They include those from cardiomyocytes, ECs, and epicardium that maintain cardiac structure; those from macrophages that regulate the cardiac inflammatory response; those from fibroblasts and RACs that promote cardiac regeneration and repair; and those from CPCs and CDCs that have proliferation and differentiation functions.

### 3.2 Mechanism of stem cell‐derived EVs in treating CVDs

#### 3.2.1 EVs derived from BMSCs

**FIGURE 3:**
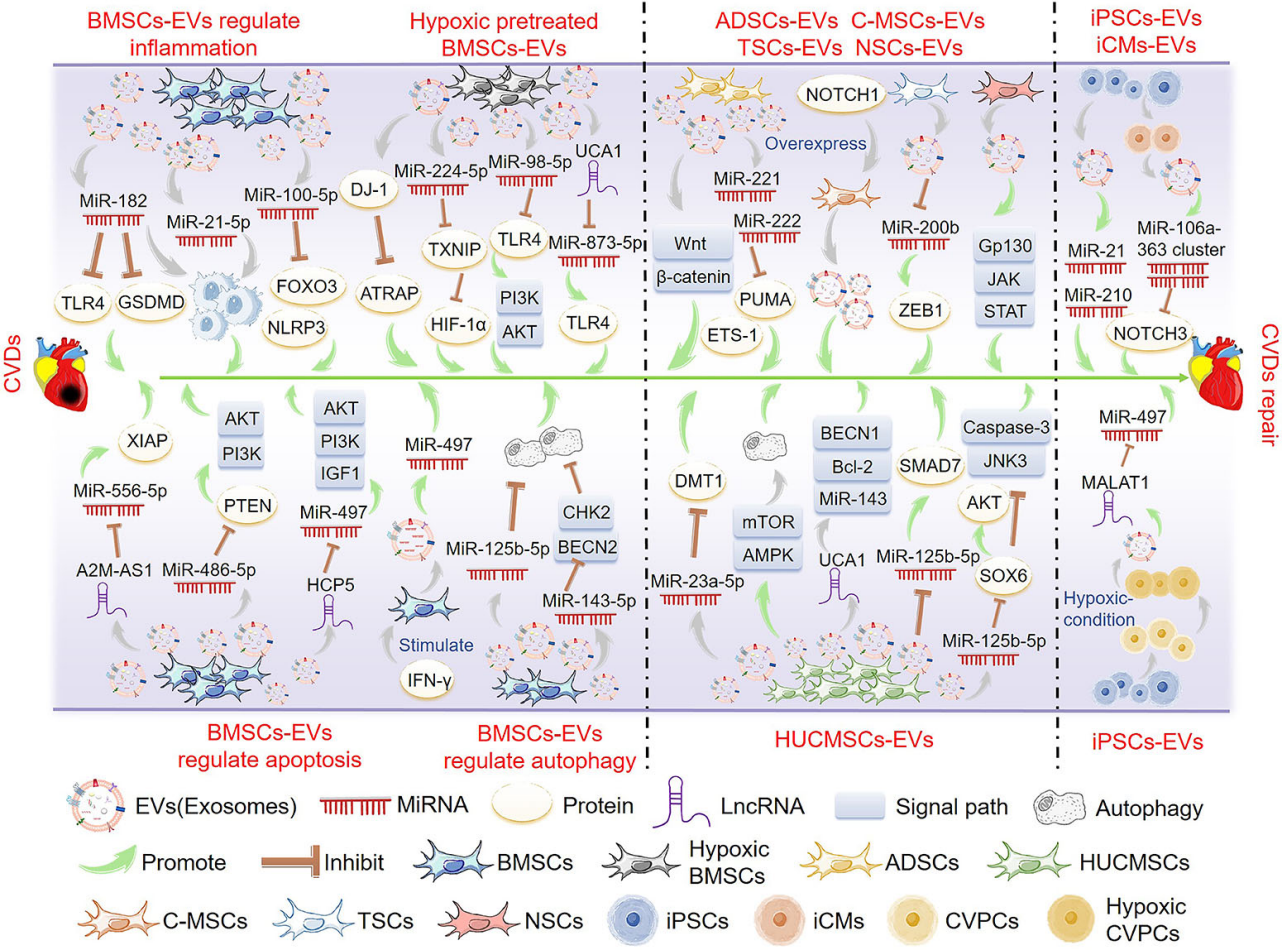
The main mechanism of extracellular vesicles (EVs) derived from different kinds of stem cells to promote the repair of cardiovascular diseases (CVDs). EVs derived from bone marrow mesenchymal stem cells (BMSCs) can promote cardiac repair by reducing inflammation, inhibiting apoptosis, and regulating autophagy. EVs derived from other types of stem cells, such as adipose tissue mesenchymal stem cells (ADSCs), human umbilical cord‐derived mesenchymal stem cells (HUCMSCs), cardiac mesenchymal stem cells (C‐MSCs), trophoblast stem cells (TSCs), and neural stem cells (NSCs) can promote CVDs repair by regulating related signaling pathways. EVs derived from induced pluripotent stem cells (iPSCs) or differentiated iPSCs promote the repair of CVDs through microRNA (miRNA) and long non‐coding RNA (lncRNA) delivery. The green arrow represents the promotion of CVDs repair.

MSCs can differentiate into ECs, heart cells, or vascular smooth muscle cells. Many studies have shown that stem cells may act by releasing EVs with cardio‐protective properties^93^ (Figure 3). BMSCs are one of the most common cell types used in cardiomyocyte therapeutic research because of their relatively easy availability, high proliferation, and anti‐inflammatory and low immunogenic properties.^94^

MSCs‐derived EVs play an important role in the progression of inflammation associated with CVDs. Studies have shown that MSC‐derived exosomes have immunomodulatory effects on macrophages in myocardial I/R injury models and inhibit *TLR4* activity mainly through the delivery of exosomal miR‐182, generating potential effects in treating myocardial I/R injury.^95^ Other studies have shown that MSC‐derived exosomes containing miR‐182‐5p ameliorated cardiac function and reduced MI in mice by targeting *GSDMD* and reducing inflammation and pyroptosis in vivo.^96^ In addition, MSC‐derived exosomes modulated macrophage polarization and suppressed inflammatory responses via miR‐21‐5p, promoting myocardial repair following reperfusion injury.^97^ Moreover, the enrichment of miR‐100‐5p in human umbilical cord‐derived MSC (hUCMSC)‐derived exosomes inhibits forkhead box O3 (*FOXO3*) expression, *NLRP3* inflammasome activation, and cytokine release, protecting cardiomyocytes from H/R‐induced pyrodeath and injury.^98^ Besides, milk fat globule‐epidermal growth factor‐factor VIII (MFGE8) in MSCs‐derived exosomes activates phagocytosis signaling, which enhances the removal of apoptotic cells and the resolution of inflammation, thus effectively repairing the heart.^99^

MSC‐derived EVs can also reduce apoptosis, promote cell proliferation, and thus reduce myocardial damage. MSC‐derived EVs can improve angiogenesis and play a therapeutic role in MI by delivering miR‐210.^100^ In addition, miR‐221 delivered by MSC‐derived EVs inhibited atherosclerotic plaque formation in atherosclerotic model mice.^101^ Other studies have found that lncRNA A2M antisense RNA 1 (*A2M‐AS1*) can reduce H/R‐induced myocardial apoptosis and oxidative stress by regulating miR‐556‐5p/X‐linked inhibitor of apoptosis (XIAP) via the delivery of MSC‐derived exosomes, regulating myocardial I/R injury.^102^ Moreover, studies have shown that the presence of miR‐486‐5p in BMSC‐derived exosomes activates the PI3K/AKT signaling pathway by suppressing *PTEN* expression, inhibiting the apoptosis of injured cardiomyocytes and protecting against myocardial ischemic injury.^103^ MSC‐derived exosomes were shown to be more effective in treating MI in rats when combined with interferon‐γ through the action of miR‐21, which promoted angiogenesis and reduced apoptosis, protecting against myocardial injury.^104^ In addition, lncRNA HLA complex P5 (*HCP5*) in human BMSC‐derived exosomes alleviates myocardial I/R injury by activating the IGF‐1/PI3K/AKT pathway by sponging miR‐497.^105^

While autophagy is a mechanism that can maintain cell viability by eliminating damaged organelles through ATP degradation under mild ischemic stress, it can exacerbate cardiac dysfunction and induce cell death when activated under severe ischemic conditions.^106^ Studies have shown that MSCs transplantation therapy improved cardiac function and infarct size in infarcted mouse hearts, primarily through exosomes containing miR‐125b‐5p, while reducing apoptosis and autophagy flux.^107^ In addition, MSC‐derived exosomal miR‐143‐3p‐regulated autophagy through the checkpoint kinase 2 (CHEK2/CHK2)/beclin 2 (BECN2) pathway, effectively reducing apoptosis and inhibiting myocardial I/R injury.^108^

#### 3.2.2 EVs derived from hypoxic preconditioned BMSCs

Subjecting heart cells to hypoxic preconditioning can improve and strengthen their ability to withstand and adjust to hypoxia, inflammation, and oxidative stress. Studies have shown that compared with EVs secreted by normoxic MSCs, EVs secreted by hypoxic‐preconditioned MSCs may better prevent myocardial I/R injury, and the direct binding of miR‐224‐5p in EVs with *TXNIP* is a key protective mechanism against myocardial cell death.^109^ EVs from hypoxic‐preconditioned MSCs have also been shown to reduce myocardial injury by targeting the *TXNIP*‐mediated HIF‐1α pathway.^110^ In addition, exosomal miR‐98‐5p from hypoxic BMSCs inhibited myocardial I/R injury by decreasing *TLR4* expression and activating the PI3K/AKT signaling pathway.^111^ Other studies have found that lncRNA urothelial cancer associated 1 (*UCA1*) in MSC‐derived exosomes secreted under hypoxic conditions provides cardiac protection through the miR‐873‐5p/XIAP axis.^112^ Moreover, cardiac hypertrophy is a pathological myocardial remodeling process in a variety of CVDs, characterized by cardiomyocyte hypertrophy, interstitial fibrosis, and decreased myocardial compliance.^113^ Parkinson disease protein 7 (PARK7/DJ‐1) contained in EVs derived from hypoxic MSCs alleviates cardiac hypertrophy by inhibiting mitochondrial dysfunction and preventing AT1R‐associated protein (ATRAP) degradation.^114^

#### 3.2.3 EVs derived from other types of stem cells

Using exosomes derived from adipose tissue MSCs (ADSCs) safeguarded rat myocardium against I/R injury via activation of the Wnt/β‐catenin signaling pathway.^115^ Moreover, studies have shown that ADSC‐derived exosomes prevent heart I/R injury via miR‐221/miR‐222 targeting the B‐cell leukemia‐lymphoma 2 (BCL2)‐binding component 3 (BBC3/PUMA)/ETS proto‐oncogene 1 transcription factor (ETS1) pathway and are effective inhibitors of I/R‐induced heart injury.^116^ In addition, ADSC‐derived exosomes prevent oxidative stress‐induced cardiomyocyte apoptosis and have cardioprotective effects against I/R injury.^117^

Human umbilical cord‐derived mesenchymal stem cell (HUCMSC) derived exosomes were shown to alleviate the damage caused by AMI.^118^ MSC‐derived exosomes from human cord blood may inhibit solute carrier family 11 member 2 (*SLC11A2*/*DMT1*) expression and iron death through miR‐23a‐3p, alleviating myocardial injury in mice with AMI.^119^ Viral myocarditis is a significant contributor to HF, arrhythmia, and sudden death in young individuals, and it is characterized by immune damage, viral infection‐induced autophagy, and apoptosis.^120^ HUCMSC‐derived exosomes can alleviate coxsackievirus B3‐induced myocarditis by activating the AMP‐activated protein kinase (AMPK)/mechanistic target of rapamycin kinase (mTOR)‐mediated autophagy flux pathway, reducing myocardial cell apoptosis and significantly alleviating myocardial injury.^121^ HUCMSC‐derived exosome transfer lncRNA *UCA1* alleviated I/R damage via the miR‐143/BCL2/BECN1 axis.^122^ In addition, hUCMSC‐derived exosomes may promote SMAD family member 7 (*SMAD7*) expression and increase myocardial injury repair by inhibiting miR‐125b‐5p.^123^ Moreover, hUCMSC‐derived exosomes protect myocardial cells from AMI damage by transferring miR‐19a to target *SOX6*, activating *AKT*, and inhibiting MAPK10/JNK3/CASP3 activation.^124^ The EVs secreted by human amniotic fluid mesenchymal stromal cells have the potential for cardiac protection and migration and can protect the heart of rats from I/R injury in vivo.^125^

Cardiac MSCs (C‐MSCs) are a novel subgroup of MSCs derived from cardiac tissue.^126^ Increasing evidence indicates that Notch signaling is reactivated during myocardial injury and triggers cardiac repair following such injury. Studies have used EVs released by notch receptor 1 (*Notch1*)‐overexpressing C‐MSCs to demonstrate their ability to prevent cell death, stimulate angiogenesis and myocardial cell proliferation, and ultimately restore cardiac function after MI.^127^

Trophoblast stem cells (TSCs) originate from monolayer blastocysts during the earliest cell differentiation. Studies have shown that transplantation of cardiac TSCs can ameliorate incorrect myocardial remodeling and improve cardiac function.^128^ TSC‐derived exosomes show anti‐apoptotic and anti‐inflammatory properties by regulating miR‐200b and its downstream target zinc finger E‐box binding homeobox 1 (*ZEB1*), alleviating DOX‐induced heart damage and offering a novel therapeutic approach for HF.^129^ In addition, exosomes secreted by neural stem cells (NSCs) have been found to protect the heart against I/R injury by activating the JAK1/2 and interleukin 6 cytokine family signal transducer (IL6ST/GP130) signaling pathways.^130^ Additional research has indicated that exosomes derived from cortical diaphyseal tissues have therapeutic potential for managing myocardial I/R injury and subsequent cardiac remodeling.^131^

#### 3.2.4 EVs derived from embryonic stem cells, induced pluripotent stem cells, and cardiovascular PCs

The enormous potential of pluripotent stem cells, such as embryonic (ESCs) and induced pluripotent (iPSCs) stem cells, to differentiate into various cardiac cell types has made them a promising candidate for cardiac regeneration.^132^ ESC‐derived exosomes have been shown to promote endogenous repair mechanisms and enhance cardiac function after MI.^133^ Additionally, EVs secreted by cardiovascular progenitor cells (Pg) derived from ESCs can effectively treat congestive HF.^134^

Other studies have shown that iPSC‐derived EVs confer protective properties on heart cells in vitro and induce angiogenesis and improve apoptosis in vivo. Due to their cell‐free nature, iPSC‐EVs are a safer alternative for treating patients with ischemic myocardial injury.^135^ In addition, one study used magnetic resonance imaging to track the homing of iPSC‐derived magnetic EVs in different animal models of myocardial ischemia. Their findings showed that iPSC‐derived EVs showed a strong tropism toward the myocardial injury site and had significant protective effects.^136^ Other studies have suggested that iPSCs in ischemic tissues showed robust proliferation and survival, and injection of iPSC‐derived exosomes into mouse ischemic myocardium could prevent myocardial I/R injury. Moreover, iPSC‐derived exosomes could provide cardiac protective miRNAs to H9C2 cardiomyocytes in vitro, including Nanog homeobox (*NANOG*)‐regulated miR‐21 and HIF‐1α‐regulated miR‐210. Therefore, iPSC‐derived exosomes represent a novel class of biological nanoparticles for treating myocardial I/R injury.^137^

Furthermore, exosomes produced by cardiac cells derived from iPSCs enhanced myocardial recovery without elevating the incidence of arrhythmic complications, providing another therapeutic option for CVDs.^138^ Studies have shown that iPSC‐derived cardiomyocytes (iCMs) could be directly applied to the injured myocardium (including in non‐human primates).^139^ In addition, the miR‐106a‐363 cluster in EVs isolated from human iPSC‐derived iCMs exposed to hypoxia and normoxia conditions promoted endogenous myocardial damage repair in ischemic heart injury through the notch 3 (NOTCH3) pathway.^140^

Mitochondria‐rich EVs (M‐EVs) were isolated from the medium of iPSC‐derived iCMs. Intramuscular injection of these M‐EVs facilitated the transfer of their mitochondrial and non‐mitochondrial cargo into injured cardiomyocytes, improving cardiac function in mouse models of MI by restoring cellular bioenergy. The proposed method shows potential as a new and accurate treatment for mitochondria‐associated diseases, including HF.^141^

Additionally, iPSC‐derived cardiovascular PCs (CVPCs) might promote the healing of MI by improving myocardial cell survival and promoting angiogenesis. The cardioprotective effect of CVPC‐derived EVs was enhanced by hypoxia regulation of CVPCs, and lncRNA metastasis‐associated lung adenocarcinoma transcript 1 (*MALAT1*) was beneficial to the cardioprotective effect of CVPC‐derived EVs by targeting miRNAs.^142^ Moreover, myocyte globules derived from iPSCs also improved the recovery of myocardial injury in mice.^143^

### 3.3 EVs from blood

#### 3.3.1 EVs from serum

In addition to cardiac and stem cells, EVs from other sources also help regulate CVDs. Serum EVs obtained from patients with acute coronary syndrome conferred protection against myocardial I/R injury in vitro and in rat hearts, dependent on STAT3 phosphorylation.^144^ Another study showed that serum exosomes obtained through ischemic preconditioning protected rat hearts from I/R injury by activating the PI3K/AKT signaling pathway.^145^ Serum‐circulating exosomes also protected AMI by regulating the miR‐190a‐3p/CXCR4/C–X–C motif chemokine ligand 12 (CXCL12) pathway^146^ (Table 2).

**TABLE 2:** Mechanisms of other extracellular vesicle (EV) sources to repair myocardial damage.

Disease	Source of EVs	Regulation mechanism	Effects	Patient or animal model	Reference
Myocardial I/R injury	Serum	*DUSP6* expression promotes STAT3 phosphorylation	Mediates the protective effects of the heart	Acute coronary syndrome patients and rat models	^144^
Myocardial I/R injury	Serum	Activate the PI3K/AKT signaling pathway	Improve heart function, reduce inflammatory cytokines production, reduce myocardial cell apoptosis, and relieve I/R injury	Ischemic preconditioning rats	^145^
Myocardial infarction	Serum	miR‐190a‐3p is downregulated in circulating exosomes, regulating the miR‐190a‐3p/CXCR4/CXCL12 pathway	It has a protective effect on myocardial injury	Acute myocardial infarction mouse model	^146^
Myocardial I/R injury	Plasma	HSP70/TLR4 axis	Delivers the signal to the heart and prevents I/R injury	Adult rats and humans	^148^
Myocardial I/R injury	Plasma	miR‐130a‐3p targets and negatively regulates *ATG16L1* gene	It can relieve excessive myocardial autophagy and improve myocardial I/R injury	I/R injury rat model	^150^
Myocardial I/R injury	Plasma	Regulation of EVs miRNAs (miR‐16‐5p, miR‐144‐3p, and miR‐451a)	Circulating EVs accumulate in injured myocardium and prevent I/R injury	Sprague–Dawley rat model	^154^
Myocardial I/R injury	Plasma	Exosomes transfer miR‐24	Reduce cell apoptosis and reduce myocardial I/R injury	Remote ischemic regulation rat	^155^
Myocardial I/R injury	Plasma	Exosomal miR‐126a‐3p	Reduce myocardial I/R damage	I/R rat model	^156^
Myocardial I/R injury	Plasma	Exosomal miR‐342‐3p targets *SOX6* and *TFEB*, respectively	Inhibit cardiomyocyte apoptosis and autophagy	Sprague‐Dawley rats	^157^
Coronary artery disease	Plasma	lncRNA AS1 in EVs is significantly upregulated and regulate the expression of VEGFA	Promote angiogenesis	Patients with coronary artery disease	^147^
Heart failure	Plasma	Ischemic preconditioning exosomes activate survival kinase in heart tissue	May have a cardiac protective effect against heart failure after myocardial infarction	Rat model of heart failure	^149^
Acute myocardial ischemic injury	Patient blood	Deletion of lncRNA *HCG15* in exosomes activates the NF‐κB/p65 and p38 pathways	Prevention of acute myocardial ischemic injury	I/R mouse model	^151^
Myocardial I/R injury	Peripheral blood	miR‐17‐3p regulates the expression of *TIMP3* in exosomes	Relief of programmed necrosis associated with I/R injury	I/R mouse model	^152^
Myocardial I/R injury	HEK293 cells	Calcium ion carrier‐induced EVs regulate *HO‐1* in a TLR4‐independent manner	Inhibition of simulated I/R damage in cardiomyocyte derived cell lines	Cells	^158^
Myocardial I/R injury	Adipocyte	EVs contain respiratory but oxygen‐impaired mitochondrial particles that regulate ROS	Protect myocardial cells from acute oxidative stress and inhibit myocardial I/R injury	I/R mouse model	^159^

Abbreviations: AKT, protein kinase B; I/R, ischemia/reperfusion; lncRNA, long non‐coding RNA; NF‐κB, nuclear factor kappa B; PI3K, phosphoinositide 3‐kinase; ROS, reactive oxygen species; STAT3, signal transducer and activator of transcription 3; TLR4, Toll‐like receptor 4.

#### 3.3.2 EVs from plasma

Studies have shown endogenous plasma exosomes also have strong cardioprotective effects in CVDs models. LncRNA AS1 in plasma EVs in patients with CAD is significantly upregulated, regulating the expression of VEGFA in ECs, thereby promoting angiogenesis.^147^ The heat shock protein 70 (HSP70)/TLR4 axis is a key pathway for exosome‐mediated cardiac protection.^148^ Other studies have shown that plasma exosomes produced by ischemic preconditioning have cardioprotective effects in rat HF models, protecting the heart in vivo.^149^ In addition, plasma‐derived EVs containing miR‐130a‐3p reduced excessive autophagy and ameliorated myocardial I/R injury through miR‐130a‐3p, which targets and inhibits the autophagy‐related gene autophagy‐related 16 like 1 (*ATG16L1)* in cardiomyocytes.^150^ Moreover, the absence of lncRNA HLA complex group 15 (*HCG15*) in the exosomes of MI patients prevented AMI via the NF‐κB/p65 and p38 pathways.^151^

The role of programmed necrosis in I/R injury is critical. Studies have shown that miR‐17‐3p in peripheral blood exosomes following cardiac I/R injury mitigated programmed necrosis and attenuated cardiac I/R injury in vivo by modulating TIMP metallopeptidase inhibitor 3 (*TIMP3*) expression.^152^ In addition, remote ischemic regulation (RIPC) protects the heart from fatal ischemic injury. EVs released from the heart after I/R were necessary for RIPC cardiac protection, demonstrating the importance of vesicular transfer mechanisms in remote cardiac protection.^153^ In addition, other studies have indicated that the beneficial impact of RIPC on the heart can be mediated through the plasma and facilitated by circulating EVs that accumulate in the damaged myocardium. The underlying mechanism involves the regulation of three miRNAs (miR‐16‐5p, miR‐144‐3p, and miR‐451a) within the EVs to prevent I/R injury.^154^ In addition, RIPC‐induced production of plasma exosomes can reduce apoptosis and myocardial I/R injury by paracrine transfer of miR‐24.^155^ Moreover, plasma exosomes in late RIPC can reduce myocardial I/R injury through exosomal miR‐126a‐3p.^156^ Furthermore, miR‐342‐3p dysregulation in plasma exosomes during early recovery after AMI may cause impaired cardiac protective potential. By targeting *SOX6* and transcription factor EB (*TFEB*), miR‐342‐3p can enhance exosome‐mediated repair of cardiac injury by suppressing myocardial apoptosis and autophagy.^157^

#### 3.3.3 EVs from in vitro cells

Transient activation of Toll‐like receptors is associated with cardiac protection, which can be achieved by blood‐derived EV therapy. However, it is difficult to isolate EVs from blood. Therefore, a cell model was established from which heart‐protective EVs could be isolated in a repeatable manner by inducing EV release by HEK293 cells using a calcium ion carrier A23187. EVs induced by a calcium ion carrier played a protective role in I/R injury by regulating heme oxygenase 1 (*HMOX1/HO‐1*) in a TLR4‐independent manner.^158^ In addition, adipocytes release small EVs (sEVs) containing respirable but oxygen‐impaired mitochondrial particles, which enter the bloodstream and get absorbed by cardiomyocytes. This mechanism shields cardiomyocytes against acute oxidative stress, and the administration of sEVs curbed myocardial I/R injury in mice.^159^

At present, EVs has become a promising treatment for CVDs. Due to the mechanism of EVs involvement in the treatment of CVDs and the limitations of its application in vivo, the modification of EVs and its combination with biomaterials have also attracted much attention.

## 4 APPLICATION OF MODIFIED EVS IN TREATING CVDS

**FIGURE 4:**
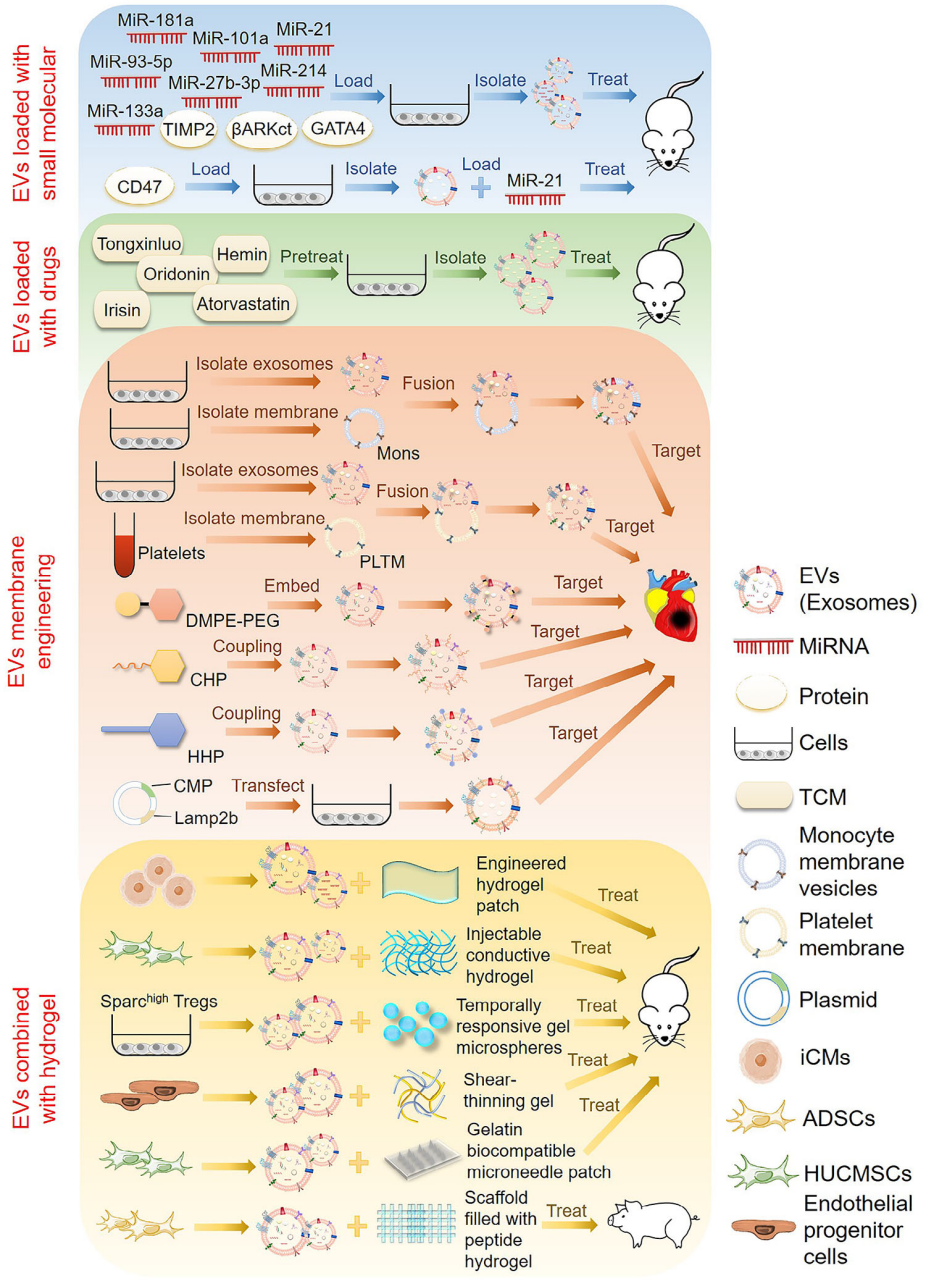
The main treatment of cardiovascular diseases (CVDs) based on extracellular vesicle (EV) modification. Therapeutic molecules such as microRNA (miRNA) and proteins were loaded into EVs to treat myocardial injury in mouse models. EVs isolated from cells pretreated with the drugs has the effect of treating CVDs. EVs modified by membrane engineering and genetic engineering can better target and treat the damaged areas of the cardiac. EVs can be combined with different kinds of hydrogels to make therapeutic cardiac patches or cardiac scaffolds, which have therapeutic effects in mouse and pig models.

As a natural carrier, EVs can transport various biomolecules to specific tissues, protect their contents from degradation, and quickly transport drug molecules to the recipient cells. EVs have good biocompatibility without causing toxicity and adverse immune reactions.^46^ In addition, the ligands on the surface of EVs achieve cell specificity by binding to the cell surface receptors on the target cells.^160^ However, the clinical therapeutic effect of simply applying EVs is still lacking. Designing and optimizing EVs and improving their therapeutic effect is necessary to make them a new clinical therapy. Overexpression of therapeutic molecular genes, loading exogenous drugs, or enhancing EV targeting through membrane engineering can improve their therapeutic efficacy (Figure 4). Combining EVs with hydrogels and other biological materials is also an important research direction for heart patches and heart stents.

### 4.1 EVs targeted delivery of therapeutic molecules for the treatment of CVDs

#### 4.1.1 EVs modified with small biomolecules

EVs have the potential to serve as a natural carrier for delivering drugs, and their effectiveness can be enhanced by incorporating bioactive molecules (e.g., miRNAs, proteins, and synthetic molecules) and engineering modifications. In one study, combining MSC‐derived exosomes with the delivery of miR‐181a had a synergistic effect, using both the immunosuppressive properties of the miRNA and the targeted delivery capability of the MSC‐derived exosomes. Mice suffering from myocardial I/R injury showed significant improvements in cardiac function and reduced infarct size after receiving treatment with MSC‐derived exosomes loaded with miR‐181a.^161^ In addition, studies have shown that miR‐101a has an anti‐fibrotic effect. MSC‐derived extracellular nanovesicles loaded with miR‐101a could be administered in a minimally invasive manner to promote recovery after heart infarction.^162^ Additionally, exosomes derived from miR‐214 overexpressed MSCs greatly enhance the therapeutic effect of AMI by promoting cardiomyocyte survival and EC function.^163^

In addition, other studies have isolated EVs with CD47‐modified membrane surfaces from MSCs overexpressing *CD47*. EVs loaded with exogenous miR‐21 had a good anti‐apoptotic effect in vitro and in vivo, which can relieve cardiac inflammation and be used to treat myocardial I/R injury.^164^ Studies have shown that miR‐93‐5p expression has a cardioprotective effect after AMI. ADSC‐derived exosomes loaded with miR‐93‐5p mitigated myocardial damage caused by AMI by targeting autophagy‐related 7 (*ATG7*) and *TLR4* to inhibit autophagy and inflammatory responses.^165^ In addition, TIMP metallopeptidase inhibitor 2 (TIMP2)‐modified exosomes from hUCMSCs enhanced the repair of MI in rats through the AKT/secreted frizzled‐related protein 2 (SFRP2) pathway, alleviating MI‐induced oxidative stress and extracellular matrix remodeling.^166^ Moreover, by upregulating miR‐27b‐3p levels in exosomes derived from hypoxia‐deficient cardiac microvascular ECs, researchers could alleviate myocardial I/R injury in rats by inhibiting FOXO1/GSDMD signaling, reducing oxidative stress, and preventing pyrodeath.^167^ Exosomes from cardiac fibroblasts overexpressing miR‐133a were also shown to inhibit myocardial pyrodeath in myocardial I/R injury, enhancing the therapeutic outcome of I/R injury.^168^ Fibroblast exosomes overexpressing *GATA4* can also play a protective role in myocardial ischemic injury.^79^ Furthermore, CDC‐derived EVs modified with βARKct, a peptide inhibitor of G‐protein‐coupled receptor kinase 2 (*GRK2*), significantly protected against ischemic injury and improved cardiac function after MI and had HF‐preventive properties in a model of HF catecholamine toxicity.^169^ Other studies have shown that genetically engineered EVs derived from HEK293T cells overexpressing miRNA‐21 can be used to treat MI and improve cardiac function.^170^

#### 4.1.2 EVs loaded with medicines

Several studies have shown that some traditional Chinese medicines, such as Tongxinluo, Oridonin, and Irisin, have therapeutic effects on CVDs. Tongxinluo pretreated MSC‐derived exosomes carrying miR‐146a‐5p significantly promoted cardiac repair through a novel mechanism targeting the interleukin 1 receptor‐associated kinase 1 (IRAK1)/NF‐κB p65 pathway and have excellent clinical translation potential.^171^ In addition, BMSC‐derived exosomes pretreated with Oridonin inhibited apoptosis by regulating autophagy activation, reducing myocardial I/R injury.^172^ Exosomes secreted by Irisin‐pretreated BMSCs alleviated the effects of myocardial scorch death and oxidative stress on myocardial H/R injury.^173^ Besides, MSCs‐derived EVs pretreated with Atorvastatin (ATV) promote macrophage polarization through the miR‐139‐3p/STAT1 pathway, significantly promoting cardiac repair after AMI.^174^ It has also been demonstrated that exosomes obtained from MSCs pretreated with ATV may significantly improve the therapeutic efficacy of AMI by upregulating lncRNA H19 and promoting ECs function.^175^ Moreover, Hemin‐pretreated MSC‐derived exosome has a protective effect on the infarcted heart, in which the exosomal miR‐183‐5p inhibits cardiomyocyte senescence by regulating the high mobility group box‐1 (HMGB1)/ERK pathway.^176^

#### 4.1.3 Membrane‐modified EVs

One major challenge to the clinical application of EVs therapy is their low yield and poor homing efficiency. To improve the delivery efficiency of EVs to injured myocardium, a membrane fusion method was used to modify MSC‐derived EVs with monocyte simulators. Imitating the recruitment characteristics of monocytes after MI enhanced the targeting efficiency of the EVs to the injured myocardium and improved cardiac function and pathologic outcomes in a mouse model of MI.^177^ In addition, immunomodulatory therapy has been identified as a promising approach for treating myocardial MI/R injury.

Studies have developed a new type of EV called platelet membrane‐modified EVs (P‐EVs), which use a membrane fusion method to mimic the binding affinity between platelets and monocytes and improve EVs targeting. In I/R myocardial injury mouse models, intravenous P‐EVs could be carried by circulating monocytes to the ischemic myocardium and release functional miRNAs into the cytoplasm, facilitating the reprogramming of inflammatory macrophages (M1 phenotype) into repair macrophages (M2 phenotype) to achieve cardiac repair by modulating the immune microenvironment.^178^ In addition, studies have shown that hybridizing exosomes with platelet membranes enhanced their ability to target injured hearts and avoid being captured by macrophages. Hybridized exosomes had a cardiac targeting ability in MI injury mouse models, enhancing their combination and accumulation in damaged tissues.^179^ Besides, an EV membrane anchoring platform called “cloaking” has also been described for directly embedding tissue‐specific antibodies or homing peptides into the EVs membrane surface in vitro to enhance their uptake by target cells. A modified glycerin–phospholipid–polyethylene glycol conjugate was added to CDC‐derived EVs by membrane engineering to target cardiac fibroblasts, myoblasts, and ischemic myocardium, showing good tissue targeting in animal models of CVDs.^180^

Studies have used a targeting peptide (cardiac homing peptide) conjugated to cardiac stem cell‐derived exosomes to target infarcted hearts with intravenous exosomes in animal models of I/R injury, significantly improving cardiac function.^181^ Other studies used CDCs to express an exosome membrane protein, lysosomal‐associated membrane protein 2 (Lamp2b), fused to a cardiomyocyte‐specific peptide (CMP). The engineered CDCs produced targeted exosomes that had the CMP on their surface. This modification increased exosome uptake by cardiomyocytes, reducing cardiomyocyte apoptosis and improving cardiac retention following intramuscular injection.^182^ This approach may be a valid strategy for evaluating the efficacy of stem cell EVs and could help manage regenerative therapies in the clinic for ischemic heart disease. Studies have also shown that exosome expressing cardiac homing peptide (HHP) on the surface preferentially targets the heart and improves the therapeutic effect of CDCs‐exosomes on cardiac hypertrophy.^183^ Moreover, the self‐assembly of a dry cell membrane‐camouflaged exosome‐mimicking nanocomplex has been reported. It recapitulates exosome function and targets ischemic cardiomyocytes to achieve efficient miRNA delivery, facilitating miRNA‐mediated myocardial repair.^184^ Other studies combined several methods to genetically modify EVs, functionalize the EV surface with cardio‐targeting peptide (CTP), and further load Curcumin and miR‐144‐3p into CTP‐EVs, thus generating cardio‐targeting EV and improving the therapeutic effect of MI.^185^

### 4.2 EVs combined with hydrogel for treating CVDs

In addition to the application of modified EVs, the application of EVs combined with biomaterials is also an exciting research direction. Bioengineering‐based therapies for CVDs often combine EVs with hydrogels. A system has been developed in which engineered hydrogel patches slowly release EVs secreted by iPSC‐derived cardiomyocytes to reduce arrhythmia load and myocardial apoptosis after infarction. The EVs are rich in cardio‐specific miRNAs known to regulate cardiomyocyte‐specific processes. Their prolonged delivery to the heart may contribute to understanding cardiac recovery mechanisms and treating cardiac injuries.^186^ Another study applied shear thinning gel (STG) to deliver EVs derived from endothelial progenitor cells, which enhanced periinfarction angiogenesis and myocardial hemodynamics after delivery to ischemic myocardium, and STG greatly improved the efficiency and efficacy of EV‐mediated therapy for myocardium.^187^

The short half‐life and rapid clearance of exosomes result in inadequate therapeutic doses for diseased areas. An injectable conductive hydrogel containing hUCMSC‐derived exosomes was developed to treat myocardial injury after I/R injury in MI. This system had a significant therapeutic effect on MI‐I/R and provided a promising therapeutic approach for damaged myocardial tissue.^188^ A gelatin‐based biocompatible microneedle patch was prepared to load exosomes isolated from hUCMSCs containing miR‐29b mimics, which can relieve inflammation, reduce infarct size, inhibit fibrosis, and improve cardiac function.^189^ Further studies were conducted to assess the effectiveness of tissue engineering techniques in delivering EVs secreted by porcine cardiac ADSCs (cATMSC‐EVs) in AMI models. They used acellular pericardial scaffolds filled with peptide hydrogels and cATMSC‐EVs purified by dimensional expulsion chromatography placed on the post‐infarction myocardium. Their results showed that EV administration enhanced cardiac function in the treated animals, indicating the potential of cATMSC‐EVs as a viable treatment option for MI.^190^

Furthermore, secreted acidic cysteine‐rich glycoprotein (*Sparc*) overexpression in Tregs might help repair infarcted tissue after AMI. The study demonstrated that EVs from *Sparc*‐overexpressing Tregs combined with temporally responsive pH/H_2_O_2_/matrix metallopeptidase 9 (MMP9) hydrogel microspheres decreased myocardial injury and enhanced cardiac function.^191^

## 5 DISCUSSION

Many risk factors accompany CVDs, which has a complex disease process and pathogenesis.^192^ Being carried by EVs is an effective way for biomolecules to regulate myocardial cell apoptosis and survival through systemic circulation.^193^ EVs can specifically induce significant changes in the pathological progression of CVDs and exert their influence by regulating gene expression. EVs derived from different cardiac or stem cells can promote or inhibit CVDs through different regulatory mechanisms. The quantity and composition of circulating EVs also serve as novel indicators of cardiovascular risk.^194^ Exploring how EVs affect the progression of CVDs is crucial for developing effective cardiac protection strategies. This review provided an overview of the current understanding of the causes and management of CVDs related to EVs and explored the primary mechanisms involved in advancing these treatment approaches.

EVs represent the next generation of therapies with strong appeal as cell‐free drugs that overcome the major hurdles of cell therapy while also serving as a versatile platform.^195^ The pathological process of CVDs is associated with extensive intercellular communication. The transfer of EVs between various cardiac cells plays a key role in the physiological pathology of CVDs.^196^ Studies have shown that EVs from heart‐related cells are rapidly absorbed by heart tissue, and there is evidence that cells preferentially take up EVs from the same tissues.^197^ Besides myocardial cells, macrophages, ECs, and fibroblasts are the primary recipients of EVs, and these cell types may mediate the cardioprotective effect.^91^ These heart‐related cell‐derived EVs may play an important role in treating CVDs.

In addition, stem cell‐derived EVs are another commonly used method for repairing CVDs. MSCs have anti‐inflammatory and regenerative functions, and most literature suggests that MSC‐derived EVs show protective activity in a variety of diseases.^198^ MSCs can differentiate into bone cells, fat cells, and chondrocytes, which are considered suitable for heart damage repair. Many studies have shown that the secretions of MSCs, mainly EVs, have better therapeutic effects than the MSCs themselves.^199^ EVs allow for easier quality control and better biosecurity. Therefore, MSC‐derived EVs are becoming a new therapeutic approach for treating CVDs.^200^ Moreover, increasing evidence shows that EVs derived from ESCs or iPSCs have a promising application prospect in treating CVDs.

While EVs have shown encouraging outcomes in managing cardiac injury, certain issues remain to be resolved. The route, time, and location of EV injection into the heart also have a certain impact on the therapeutic effect of EVs. The application of EVs can be optimized by enhancing the targeting of EVs to the heart muscle. However, it is difficult to determine the specific amount of EVs administered in preclinical trials since it needs to be adjusted under different conditions and models.^15^ In addition, certain challenges exist in isolating and characterizing EVs, necessitating further investigation into extraction techniques to enhance the quantity and quality of EVs obtained. Furthermore, different EV populations can be obtained by different separation methods, such as the ultrafast centrifugal or kit methods.^201^ Whether these differences lead to inconsistent outcomes in EV treatment remains unclear. There is a need to establish uniform standards for EV extraction and quantity to better assess their application value.^202^

The low application efficiency of EVs is the main obstacle to their clinical use. However, researchers are making progress in addressing these issues as well. Compared with directly extracted EVs, artificial EVs loaded with biomolecules can achieve better therapeutic effects at the genetic level and can also reverse the regulatory pathways leading to CVDs. Transgenic EVs have shown promising potential in treating myocardial injury. For example, overexpressing beneficial miRNAs related to myocardial damage (miR‐181a, miR‐27b‐3p, miR‐214, and miR‐133a) or loading EVs with exogenous molecules (miR‐93‐5p and miR‐21) showed better therapeutic effects than unmodified EVs. Moreover, previous studies have administered EVs via both intracoronary and intramyocardial injections. Intramyocardial administration is the more effective method, but its application in clinical settings is challenging.^203^

In addition, most intravenous EVs are absorbed in the liver, necessitating a larger EV dose for treatment. Therefore, improving EV targeting to the myocardial injury area is crucial. Several studies have modified EVs using membrane fusion methods or the expression of CTPs conjugated on their surface, significantly overcoming the problem of poor targeting efficiency and enhancing EV accumulation in the injured myocardium.^204^ Currently, studies are using synthetic biomimetic sEVs for targeted drug delivery to improve drug delivery efficiency, which may be based on EV treatment of CVDs and a promising research direction.^205^

In addition, another approach is to optimize the delivery mode of EVs. Applying biomaterial hydrogels can significantly optimize EV retention, forming stable gel patches or scaffolds on the heart and improving treatment efficacy in promoting myocardial injury in animal models. In addition, using drug‐pretreated MSC‐derived EVs may also promote therapeutic efficacy. The EVs derived from MSCs pretreated with Tongxinluo, Oridonin, Irisin, Curcumin, ATV, and hemin had enhanced therapeutic effects on CVDs. Bioengineered EVs loaded with single or multiple biomolecules and drugs are gradually becoming the favored therapeutic approach for CVDs, with advantages such as low cytotoxicity, high specificity in targeting damaged tissues, and immune escape.^206^ In conclusion, it is necessary to develop safer and more effective EV therapies for CVDs that can be applied clinically, increase the validation of EVs in animal models and clinical trials, and seek more promising research directions for treating CVDs.

## 6 CONCLUSIONS

In vitro and in vivo studies have effectively demonstrated the enormous potential of EVs in cardiology. This review summarized the specific regulatory mechanisms of EVs in the pathogenesis and treatment of CVDs. It also illustrated the impact of heart and stem cell‐derived EVs on CVDs repair and cardiac function restoration in animal models. Bioengineering of EVs can further improve their targeting and better deliver therapeutic molecules, overcoming the limitations of applying EVs. This review provided comprehensive insight into the pathogenesis and treatment of CVDs using EVs, which can provide a specific foundation for inspiring more valuable research directions.

## AUTHOR CONTRIBUTIONS

*Conceptualization*: Q.L., G.G., and D.W. *Investigation*: Q.L. and D.W. *Project administration*: G.G. and D.W. *Resources*: D.W. *Supervision*: G.G. and D.W. *Visualization and writing—original draft*: Q.L. *Writing—review and editing*: Q.L., Q.F., H.Z., C.L., X.S., C.M., L.S., G.G., and D.W. All authors have read and approved the final manuscript.

## CONFLICT OF INTEREST STATEMENT

The authors declare they have no conflicts of interest.

## ETHICS STATEMENT

Not applicable.

## Data Availability

The data that support the findings of this study are available in the review.
